# Clarifying the Mechanism of Superantigen Toxicity

**DOI:** 10.1371/journal.pbio.1001145

**Published:** 2011-09-13

**Authors:** John D. Fraser

**Affiliations:** The Maurice Wilkins Centre for Molecular Biodiscovery and School of Medical Sciences, University of Auckland, Auckland, New Zealand

Superantigens are bacterial proteins that generate a powerful immune response by binding to Major Histocompatibility Complex class II molecules on antigen-presenting cells and T cell receptors on T cells. A recent article reveals that at least one of the superantigens, staphylococcal enterotoxin B (SEB), also binds the co-stimulatory molecule CD28, suggesting that a much larger and potentially more stable complex is formed at the immunological synapse than was previously thought. This revelation greatly clarifies some of the mystery surrounding how and why these toxins are able to elicit such a toxic immune response at extremely low concentrations. These findings also highlight a novel role for CD28 in microbial pathogenicity.

## 

Bacterial superantigens (Sags) constitute a family of very stable bacterial proteins that are the most potent known activators of the immune system. They can cause food poisoning or, if they occur at sufficient concentration in the blood or lymphoid tissue, systemic shock [1]. Those unfortunate enough to eat food contaminated with *Staphylococcus aureus* will experience a brief but violent episode of vomiting and diarrhoea just a few hours later—the gut's attempt to expel the Sag before it wreaks havoc with the immune system. If a Sag does get into the bloodstream, and if the patient has no neutralising antibody from previous exposure, then the Sag will induce a sudden and profound T cell stimulation that generates a cascade of cytokines, resulting in symptoms that include high fever, headache, vomiting, hypotension, aches, and rash, causing the condition known as Toxic Shock Syndrome. This life-threatening illness is often associated with young females who have developed an intra-vaginal infection of a staphylococcal strain producing the Sag Toxic Shock Syndrome Toxin (TSST) [2],[3]. Deep tissue infections by *Streptococcus pyogenes* can also produce similarly powerful Sags capable of causing lethal shock [1]. Interestingly, the Sag-induced immune response is not targeted at the bacteria themselves, but rather Sags function to direct a nonspecific T cell- and cytokine-mediated immune response that somehow assists in bacterial survival. Although many cytokines are produced in response to a single Sag, acute toxicity is blamed on the excessive production of three T cell cytokines—Interleukin-2 (IL-2), Interferon-γ (INF-γ), and particularly Tumour necrosis Factor α (TNF-α) [4],[5].

Perhaps the most notable feature of Sags is their extreme potency: many of the more than 30 different staphylococcal and streptococcal Sags stimulate profound proliferation and cytokine production in up to 20% of all peripheral T cells, at concentrations in culture that approach 1 femtomolar (10^−15^ moles/l). This is especially remarkable since T cells are not directly involved in the immediate defence against these bacteria. Why *S. aureus* and *S. pyogenes* should produce such a powerful T cell response has never been clearly resolved. One hypothesis proposes that Sags are important in the very early stages of colonisation when the bacteria are struggling to establish a niche. By stimulating local T cells, Sags may suppress the recruitment and activation of their real enemy—neutrophils and macrophages, which would destroy the bacteria. Sags must therefore be effective at vanishingly low concentrations and it is only when the bacterial colony becomes well established and fails to shut off Sag production that the toxic *sequelae* arise—a state that can be of little benefit to the bacteria and even less benefit to the host.

Normally, microbial antigens are internalized by antigen presenting cells, digested, and then presented as small peptides on the cell surface bound together with Major Histocompatability Complex class II (MHC class II) molecules; the combination of MHC and peptide is then recognized by T cell Receptors (TcRs) expressed on T cells, thus stimulating an immune response specific to that peptide antigen. However, over the past two decades many elegant studies have revealed that all Sags do one thing very well—they hijack T cell antigen recognition by directly cross-linking MHC class II and TcR, thus bypassing the antigen-presenting stage and stimulating a much larger, inappropriate immune response. All Sags therefore have at least two separate binding sites—one for MHC class II and another for the β-chain of TcR [6]. Mutagenesis studies have mapped these sites for a number of different Sags and co-crystal structures of Sags bound to either MHC class II or TcR have confirmed their location [7],[8]. What has been most surprising is the variety of binding modes used by individual Sags (Figure 1). For example Staphylococcal enterotoxin B (SEB) and C (SEC) cross-link MHC class II α-chain and TcR β-chain. Streptococcal pyrogenic exotoxin C (SPEC) binds to the other side of MHC class II and cross-links TcR β-chain while Staphylococcal Enterotoxin A (SEA) binds both α- and β-chain binding sites on MHC class II to cross-link TcR β-chain. Staphylococcal enterotoxin H (SEH) is the only Sag that binds to a TcR α-chain (Figure 1) [9]. Thus although Sags share a very similar protein structure, each has evolved its own way of binding to MHC and TcR—and this remarkable binding diversity clearly offers *S. aureus* and *S. pyogenes* an important survival advantage.

**Figure 1:**
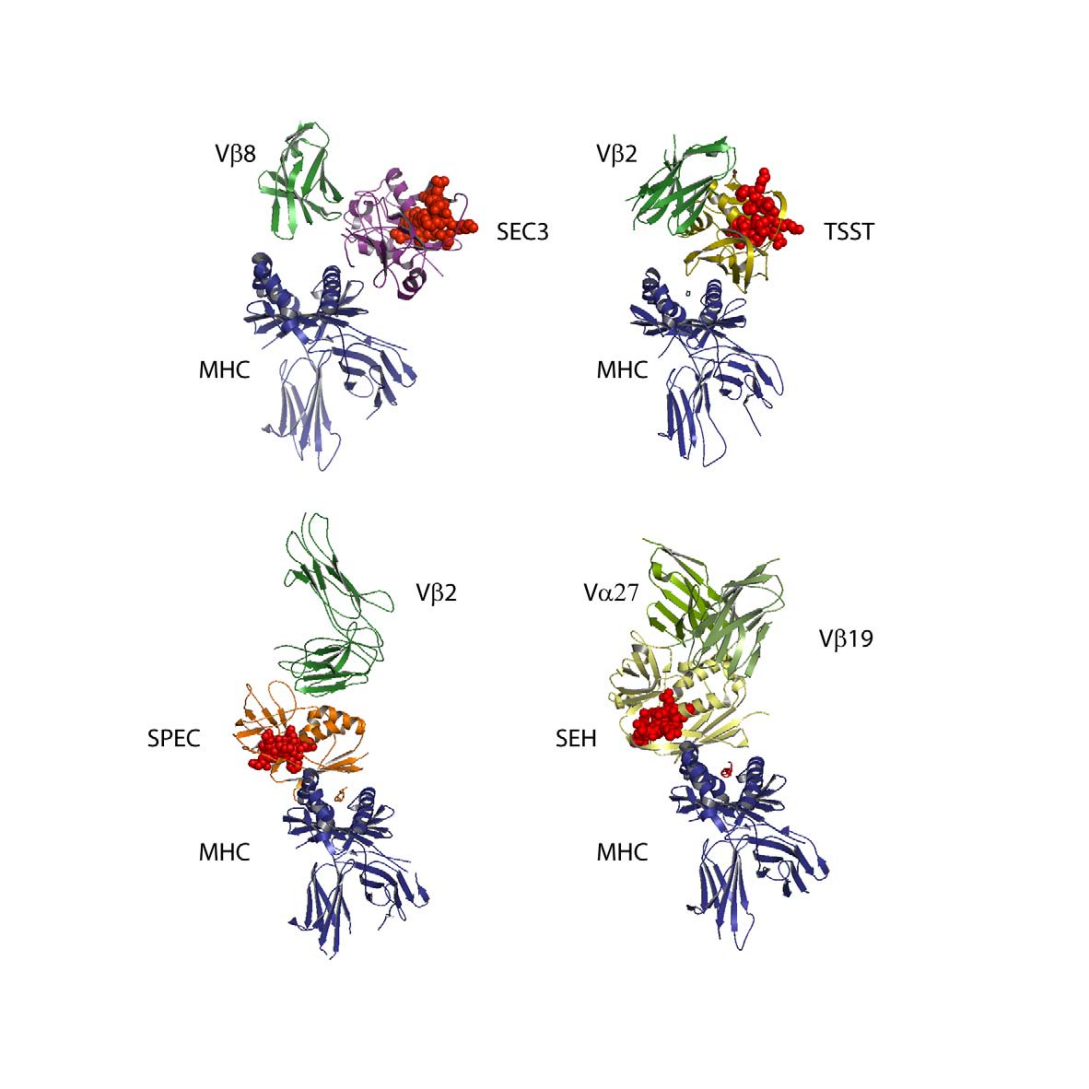
The model structures of four trimer complexes reveal the diversity in Sags binding. In the first structure, SEC3 (similar to SEB) is bound to the conserved MHC class II α-chain and engages a Vβ8 domain [18],[19]. Note how the TcR makes no contact with the normal peptide/MHC surface as it would during normal peptide recognition. In the second structure TSST binds in the same location as SEC3 but in a different orientation. TSST is very specific for the human Vβ2 TcR [20],[21]. In the third structure, the streptococcal Sag SPEC is bound to the other side of MHC class II and ligates human Vβ2 TcR [8],[22] but in a quite different orientation to TSST. The fourth structure is of staphylococcal enterotoxin H (SEH) bound to the β-chain of MHC class II but ligating a TcR through its Vα27 domain—the only Sag known to engage a TcR α-chain [9]. In each of the structures the location of the CD28 binding identified by Arad et al. [16] is represented by red space filling spheres. Note its position well away from both TcR and MHC class II in a suitable position to engage a CD28 molecule in a tetrameric membrane complex in each of the four Sag orientations.

Although this Sag-MHC-TcR trimer model of Sag activation is universally accepted [6], there are several perplexing aspects of Sag behaviour that have never quite gelled, hinting that something else might better explain their extreme potency and toxicity. For example, the binding affinities that Sags display towards TcR and MHC class II are typically quite weak—in the low micromolar range, yet Sags stimulate at concentrations several million times lower than this [1]. Sags are also several orders of magnitude less potent towards mouse T cells than human T cells, even when presented on human MHC class II [10]—a discrepancy that has never been fully explained. Mutating the TcR binding site on Sags creates mutants that have no mitogenicity yet still retain significant toxicity especially when combined with small amounts of the Gram negative bacterial pyrogen Lipopolysaccharide (LPS). This is a particularly lethal combination, suggesting that mitogenicity and toxicity may utilize separate mechanisms [1]. Finally, full activation and cytokine production demands that T cells receive a second co-stimulatory signal through the CD28 molecule, yet there was no evidence that CD28 was effectively engaged during Sag activation. Even so, T cells defective in CD28 are much less responsive to Sag and CD28 deficient mice are completely resistant to Sag toxicity, producing no TNF-α or INF-γ [11],[12]. It is well-known that CD28 on its own can deliver a potent signal to T cells. This is best highlighted by the unfortunate 2006 clinical trial of the company TeGenero's humanised anti-CD28 monoclonal antibody TGN1412: unexpectedly all six healthy volunteers suffered near fatal toxic shock immediately following injection of a small amount of TGN1412 [13]. In an insightful letter in the *New England Journal of Medicine* discussing this unfortunate incident, David Corry and Dorothy Lewis from the Baylor College of Medicine proposed a linkage between the anti-CD28 toxicity observed in this trial and Sags: “… together these observations indicate that T cells activated by superantigen binding to both CD28 and antigen receptor mediate the toxic shock syndrome” [14],[15]. New evidence, presented in this issue of *PLoS Biology*, suggests they were right!

A paper in this issue of *PLoS Biology* [16] has carefully re-examined the Sag staphylococcal enterotoxin B (SEB) and follows on from an earlier study that first identified peptide antagonists of SEB toxicity [14]. SEB is best known for its food poisoning abilities, and although it is a relatively weak human T cell mitogen compared to other Sags, it produces large amounts of IL-2, TNF-α, and INF-γ when added to human peripheral blood cells and kills mice that have been pre-sensitised with the liver toxin D-galactosamine [17]. In addition to MHC class II and TcR, Arad et al. [16] found that SEB possesses a third binding site to—you guessed it—CD28. This is a revelation and in hindsight makes perfect sense, providing a much clearer rationale for the extreme potency and toxicity exhibited by SEB and possibly other Sags. These findings also highlight a novel role for CD28 in microbial pathogenicity. The authors show that SEB binds to a soluble form of CD28 through a relatively conserved region that is distinct from both the MHC class II binding site and the TcR binding site (shown by the red spheres in Figure 1) [14]. They use short synthetic peptides that mimic both the SEB and CD28 regions to effectively block SEB-induced cytokine production by T cells. To strengthen their case, the authors mutate the predicted CD28 binding site on SEB and show that this mutant fails to stimulate IL-2, TNF-α, and INF-γ in T cells. SEB is also shown to bind to cells transfected with CD28 that lack either MHC class II or TcR. Finally they use the technique of peptide phage display to generate novel synthetic peptides screened for inhibiting SEB binding to CD28-Fc. These peptides, as well as peptides derived directly from CD28, protect mice from SEB-induced lethality. Importantly the authors show that the same CD28 binding site can be found on other Sags such as SEA and TSST and that the synthetic peptides that inhibit SEB also inhibit SEA- and TSST-induced cytokine production [14]. This suggests that CD28 is a general target for all bacterial Sags. On the opposing side of the complex, the SEB binding site on CD28 is mapped to a region needed for homo-dimerisation that is distinct from the B7 binding site. CD28 may therefore be bound by both SEB and B7 in the resulting membrane complex (Figure 2).

**Figure 2:**
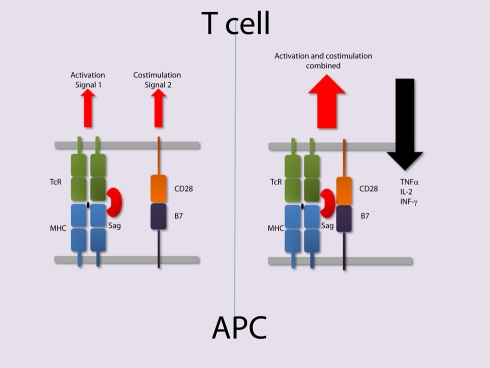
A cartoon of what might be happening at the immunological synapse during Sag engagement. In a model consistent with the crystal complexes shown in Figure 1, CD28 provides the essential 2nd co-stimulatory signal through separate B7 ligation. CD28 is essential for Sag toxicity [11],[12]. In the new model proposed on the right, CD28 is part of a larger more stable complex directly ligated by Sag. This new model explains much better the extreme potency and cytokine toxicity observed with Sags. In theory this tetrameric complex would be more stable being held together by 3 interactions rather than 2.

While this discovery greatly clarifies the mechanism of Sag toxicity, there are some nagging questions arising from this study that will need further work. The linear region identified—SEB_150–161_—is predominantly a loop structure that connects the β8-strand to the first bit of the highly conserved α4 helix situated at the core of all staphylococcal and streptococcal Sags. The 14-mer region used in these studies—VQTNKKKVTAQELD—is reasonably conserved in other Sags and is located on a face distinct from the MHC class II and TcR binding sites (Figure 1). The problem is that the majority of amino acid side chains in this sequence are buried within the native SEB structure with only 4 of the 14 side-chains exposed to the solvent and thus available to contact CD28. At the moment it's difficult to see how a linear synthetic peptide of this region can block CD28 binding when the structure it competes with is not solvent accessible. Another curious finding is the functional mutant SEB-T150A.K152A, which fails to induce cytokines in T cells but actually binds to soluble CD28 with higher affinity than native SEB. This is the opposite of what would be expected but could be explained by a model where optimal signalling through CD28 requires only transient interaction with SEB. The authors have focused only on cytokine gene expression and protection from lethal toxic shock as the means of assessing SEB activation. It would have been nice to see whether T cell proliferation is also affected to the same extent as cytokine production. If it is not, then this would suggest that T cell proliferation is primarily mediated through TcR ligation while the excessive cytokine toxicity results from CD28 ligation.

Like any revealing discovery, more questions are raised than answered—questions that will certainly be addressed in future studies. There is no doubt that a new model that places Sags in the middle of a large membrane complex consisting of MHC class II, TcR, and now CD28 will be extensively tested in the coming months. At this stage however, one can only marvel at the extraordinary efficiency of these small protein toxins to engage the three most crucial molecules in T cell antigen recognition. Exactly why they do this is the next question, but given this new CD28 connection, there are now new approaches for developing therapeutics against toxic shock.

## Abbreviations

MHC: Major Histocompatability Complex
Sag: superantigen
SEB: staphylococcal enterotoxin B
TcR: T cell Receptor
TNF-α: Tumour necrosis Factor α
TSST: Toxic Shock Syndrome Toxin
